# Identifying Regions of the Genome Associated with Conception Rate to the First Service in Holstein Heifers Bred by Artificial Insemination and as Embryo Transfer Recipients

**DOI:** 10.3390/genes15060765

**Published:** 2024-06-11

**Authors:** Victoria C. Kelson, Jennifer N. Kiser, Kimberly M. Davenport, Emaly M. Suarez, Brenda M. Murdoch, Holly L. Neibergs

**Affiliations:** 1 Department of Animal Sciences, Washington State University, Pullman, WA 99163, USA; victoria.kelson@wsu.edu (V.C.K.); kimberly.davenport@wsu.edu (K.M.D.); emaly.suarez@wsu.edu (E.M.S.); 2 Washington Animal Disease Diagnostics Laboratory, Pullman, WA 99164, USA; jennifer.kiser@wsu.edu; 3 Department of Animal, Veterinary and Food Sciences, University of Idaho, Moscow, ID 83844, USA; bmurdoch@uidaho.edu

**Keywords:** cattle, conception rate, dairy, embryonic loss, loci

## Abstract

Heifer conception rate to the first service (HCR1) is defined as the number of heifers that become pregnant to the first breeding service compared to the heifers bred. This study aimed to identify loci associated and gene sets enriched for HCR1 for heifers that were bred by artificial insemination (AI, *n* = 2829) or were embryo transfer (ET, *n* = 2086) recipients, by completing a genome-wide association analysis and gene set enrichment analysis using SNP data (GSEA-SNP). Three unique loci, containing four positional candidate genes, were associated (*p* < 1 × 10^−5^) with HCR1 for ET recipients, while the GSEA-SNP identified four gene sets (NES ≥ 3) and sixty-two leading edge genes (LEGs) enriched for HCR1. While no loci were associated with HCR1 bred by AI, one gene set and twelve LEGs were enriched (NES ≥ 3) for HCR1 with the GSEA-SNP. This included one gene (*PKD2*) shared between HCR1 AI and ET services. Identifying loci associated or enriched for HCR1 provides an opportunity to use them as genomic selection tools to facilitate the selection of cattle with higher reproductive efficiency, and to better understand embryonic loss.

## 1. Introduction

Approximately one third of dairy cattle in the US are culled due to infertility [1]. Infertility is most common in the embryonic stage, defined as that period between conception and gestation day 42, with embryonic loss accounting for up to 57% of all pregnancy losses [2,3,4]. The fertilization rates in *Bos taurus* cattle fall between 90 and 100%; however, only 60% remain pregnant by day 30 of gestation [5,6,7,8]. Pregnancy loss is financially significant to producers, resulting in USD 725 to USD 1030 per cow lost from increased inseminations, maintenance, and loss of days of milk production [9,10,11,12]. Decreasing conception rates and the significant financial impact on producers has led to research focusing on breeding and genetic-based strategies to increase fertility. The addition of genetic components associated with fertility traits such as heifer conception rate to the first service (HCR1) to producer-used selection indices has increased reproductive efficiency by 5% since being implemented in 2010 [13,14,15]. The inclusion of HCR1 in current indices does not take into account artificial insemination (AI) and embryo transfer (ET) differences, making this an area of necessary research.

Dairy cattle are primarily bred by AI; however, some animals are recipients for ET with embryos from elite parents [16]. These breeding services may include various protocols of timed hormone injections to facilitate ovulation for AI or to mimic the estrous stage of the ET donor [17,18]. Animals that are ET recipients have an in vitro fertilized embryo inserted into their uterus around day 7 of embryonic development, after which anticipated estrus, or signs of estrus, are apparent in the recipient [19].

Many studies suggest that there are important biological differences between in vitro and in vivo embryonic development. For example, there is an increased rate of blastulation for in vitro-produced embryos compared to AI or naturally serviced animals [20,21]. The use of in vitro-produced embryos and, to a lesser extent, in vivo-derived embryos, which are transferred to recipients, has been shown to lead to an increase in abnormal placentation when compared to in vivo-produced embryos that are not transferred [22]. There is also a difference in the viability of in vivo- and in vitro-produced embryos, with in vitro-produced embryos having up to 40% higher rate of mortality [23]. Many aspects can affect the success of in vitro-produced embryos; however, biological differences such as the lower likelihood of elongation and slight differences in vascularization of the yolk sac to support early embryogenesis have been shown to differ between in vivo and in vitro embryos [23,24,25]. Due to the biological differences among AI and ET breeding services, heifers bred by AI and those that served as ET recipients were analyzed separately for HCR1. This study aimed to identify loci and gene sets associated with HCR1 for animals bred by AI, or that were ET recipients. Identifying regions associated or enriched for HCR1 allows for the possible validation of fertility-related loci, positional candidate genes, and leading-edge genes. Validated loci associated with HCR1 may be incorporated into genomic selection for fertility traits and provide insights into the mechanisms of embryonic loss for heifers bred by AI or that serve as ET recipients.

## 2. Materials and Methods

### 2.1. Study Animals and Phenotype

The studied heifers (*n* = 5750) were from a single dairy in Idaho. This study was exempt from Institutional Animal Care and Use approval as all genotyping was done prior to study initiation and genotyping was initiated by the owner of the dairy. No additional handling or records were required for this study beyond those used by the dairy for management purposes. 

Dairy Comp 305 version 23.11 (Valley Agricultural Software, Tulare, CA, USA) breeding and health records were evaluated for all heifers. Animals without complete records were removed (*n* = 826). Cattle that showed health events (mastitis, lameness, or metabolic issues) 21 days prior to breeding through 30 days post-breeding were removed (*n* = 9) from the study. The exclusion of health events was performed to minimize environmental effects of infertility and enhance the genetic effects on embryonic loss.

Pregnancy was determined by ultrasound approximately 30 days post-breeding. Heifers were bred by AI or served as a recipient for ET one time per estrous cycle. Heifers served exclusively as ET recipients (*n* = 2086) or were exclusively bred by AI (*n* = 2829) (Table 1). Cattle bred by AI were inseminated following observed estrus, while cattle receiving ET were synchronized using a double ovulation synchronization protocol. HCR1 for heifers bred by AI was 56% (1575/2829) and 47% (988/2086) for heifers that served as ET recipients. Phenotypes were binomial for HCR1 for cattle bred by AI and for cattle that were ET recipients. Heifers pregnant at gestation day 30 were coded as a 1 and heifers that were not pregnant at gestation day 30 were coded as a 0 for HCR1.

### 2.2. DNA Extraction and Genotyping

Heifer genotypes were obtained from Zoetis CLARIFIDE^®^ Plus (Zoetis Precision Animal Health, Parsnippany, NJ, USA) and imputed to 634,433 SNPs using Beagle [26] and the ARS-UCD 1.2 assembly “http:/bovinegenome.org (accessed on 8 January 2024)”. The reference population for imputation consisted of approximately 4800 US Holsteins from California, New Mexico, Oregon, and Washington that were genotyped with the Illumina BovineHD BeadChip (San Diego, CA, USA). Genotyped animals shared between 29,741 and 53,594 SNPs with the BovineHD BeadChip, resulting in an imputation genotype accuracy of >95%. Imputed genotypes were then used for the genome-wide association study (GWAS) after quality control filtering.

### 2.3. Quality Control

Quality control filtering resulted in removing imputed genotypes if the call rate < 0.9 (*n* = 21,760), the individual had a minor allele frequency < 0.01 (*n* = 100,910), or if they failed Hardy–Weinberg equilibrium testing (*p* < 10 × 10^−100^, *n* = 11,100). After the completion of quality control filtering, a total of 4915 heifers and 500,663 SNPs remained for the final analysis.

### 2.4. Genome-Wide Association Study (GWAS)

A GWAS was performed for HCR1 using the SNP and Variation Suite (SVS) software version 8.1 (Golden Helix, Bozeman, MT, USA). The GWAS utilized the efficient mixed-model association eXpedited statistical approach by using an identity-by-state matrix (EMMAX-IBS). The statistical model for EMMAX is expressed as y =Xβ+Zu+ϵ; here, y = the vector of observed phenotypic values, X = a matrix containing fixed effects, *β* = regression coefficients, Z = a matrix containing the observed random effects, u = vector of random effects concerning variants of allele substitutions in the population, and ϵ = residual effects [27]. Due to the unknown inheritance of HCR1 in cattle, three genotypic models (additive, dominant, and recessive) were analyzed for each group. The additive model assumed that two minor alleles (aa) resulted in twice the effect that a single minor allele (Aa) would have on fertility. The dominant model evaluated an association with (AA/Aa) compared to (aa) alleles. The recessive model compared the presence of (AA) to (Aa/aa) genotypes. The threshold for an association was measured using the recommendation of the Wellcome Trust for unadjusted *p*-values [28].

**Figure 1:**
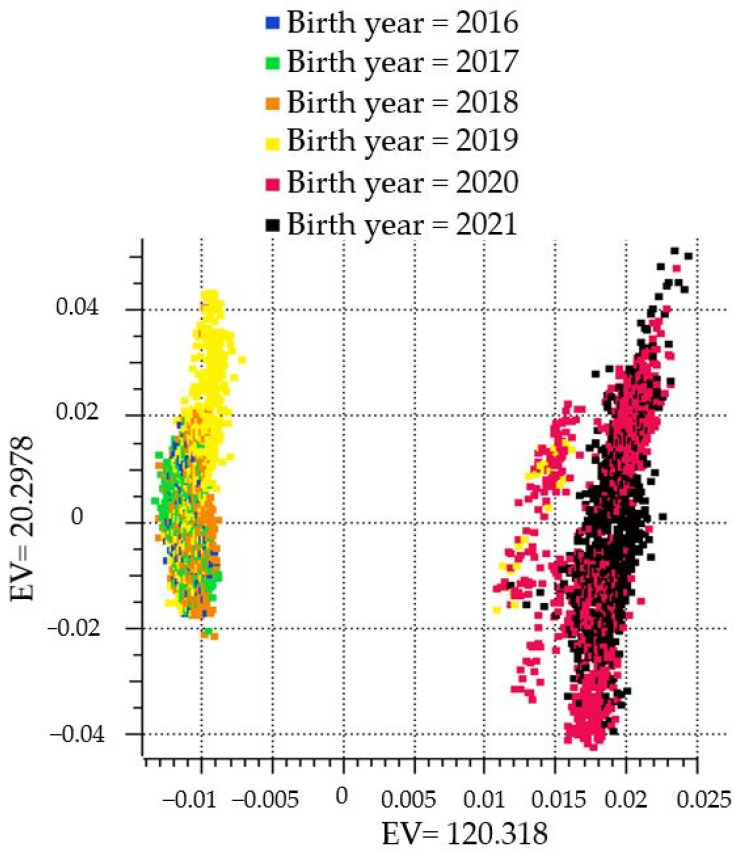
A principal component analysis (PCA) colored by birth year showing the two distinct clusters within the population.

A principal component analysis (PCA) was performed, showing two distinct clusters within the population (Figure 1) based on birth year. The distinct separation of the population into two clusters was subsequently used as a covariate to account for population stratification for the GWAS. To identify loci when several SNPs were associated with HCR1 that were in close proximity, a linkage disequilibrium threshold of D′ > 0.7 was used. This threshold was previously used to characterize loci for humans and cattle [29,30]. The genomic inflation factor lambda (λ_GC_) was calculated as described by Devlin and Kathryn Roeder in 1999 [31] for heifers that were bred by AI, and was 0.99, 0.99, and 1.02, for the additive, dominant, and recessive models, respectively. The λ_GC_ for HCR1 for heifers that were ET recipients was 1.01 for the additive and dominant models and 1.03 for the recessive model. 

The average haplotype size for the study population was 29.9 kb using the method of Gabriel et al. (2002) [32] within SVS. This haplotype size was used to identify positional candidate genes and SNPs for the gene set enrichment analysis using SNPs as gene proxies (GSEA-SNP). Genes that were within 29.9 kb from the associated SNP in the 5′ or 3′ directions were identified as positional candidate genes or as potential gene proxies for the GSEA-SNP. Locations of genes and SNPs were based on the bovine ARS-UCD 1.2 genome assembly. 

### 2.5. Gene Set Enrichment Analysis–Single Nucleotide Polymorphism

The GSEA-SNP was completed using the GenGen version 1 package in R version 3.6.3 [33]. Genes were represented by the single SNP that was the most significantly associated with HCR1 within the calculated average haplotype of the gene. Approximately 34,450 genes were evaluated within gene sets of five different databases: Gene Ontology (GO), Reactome (R), Bio Carta, Kyoto Encyclopedia of Gene and Genomes (KEGG), and Protein Analysis Through Evolutionary Relationships (PANTHER). These five databases focus on collecting data on biological pathways (gene sets) in humans and other animals that have been proven to interact during various biological processes. These processes include genes involved with genetic information, cellular processes, drug processing, connection to disease, and metabolism. Although there is less information on gene processes in cattle, these gene sets serve as a model for the relationship of genes present in cattle. SNPs were then ranked by their enrichment to HCR1 in heifers receiving AI or ET services within each gene set.

A running sum statistic was used to calculate enrichment scores for every gene set based on SNP ranking for HCR1 for AI-bred and ET-recipient heifers. Enrichment score statistics were calculated similarly to Kolmogorov–Smirnov’s weighted statistics [34]. Genes in a gene set that were enriched for HCR1 resulted in higher enrichment scores, while those that were not enriched (and were ranked lower) had lower enrichment scores. Leading-edge genes are genes within a gene set that contributed positively to the peak enrichment score for HCR1. Using the GenABEL R version 3.6.3 package, permutated *p*-values were calculated for 10,000 phenotype-based permutation tests and used for each gene set [35,36]. The enrichment scores were then normalized (NES) to account for the number of genes within the gene set. Enriched gene sets for HCR1 were defined as those with NES ≥ 3.0, which is comparable to previous fertility studies [37,38]. 

## 3. Results

### 3.1. Genome-Wide Association Analysis

**Figure 2:**
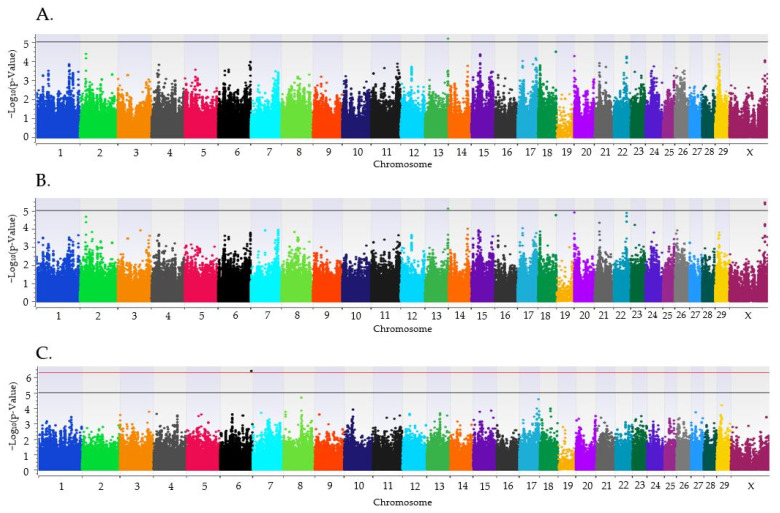
Loci associated with conception to the first service in Holstein heifers that were ET recipients for additive (**A**), dominant (**B**) and recessive (**C**) models. The axes for all plots have the *Bos taurus* chromosome on the x axis, and the −log10*p*-value on the y axis. The black line signifies the threshold for a moderate association with an uncorrected *p* < 1 × 10^−5^, and a red line indicates the threshold for a strong association (*p* < 5 × 10^−7^) with HCR1.

Three loci were associated (*p* < 1 × 10^−5^) with HCR1 for heifers that were ET recipients (Figure 2). A locus on BTA13 was associated (*p* = 6.6 × 10^−6^) with HCR1 with the additive model, BTAX was associated (*p* = 1 × 10^−6^) with HCR1 with the dominant model and BTA6 was strongly associated (*p* = 3.8 × 10^−7^) with HCR1 in the recessive model (Table 2). Positional candidate gene *AFAP1* was located on the BTA6 locus, while *ACE2*, *BMX*, and *PIR* were found within the locus on BTAX. The SNPs associated with HCR1 in the *AFAP1*, *ACE2*, and *BMX* genes were identified within the intron. No loci were associated with HCR1 for additive, dominant, or recessive models in heifers bred by AI.

### 3.2. GSEA-SNP Results

The GSEA-SNP identified one GO gene set enriched (NES = 3.0) with HCR1 for heifers bred by AI. This gene set, sodium ion transmembrane transporter activity (GO:0015081), included 13 unique leading edge genes (Table 3). The GSEA-SNP for HCR1 for ET recipients identified four GO gene sets and 66 leading edge genes (Table 3). Of the leading edge genes, glycosylphosphatidylinositol specific phospholipase D1 (*GPLD1*) was found in all four enriched gene sets, and polycistin-2 (*PKD2*) was shared between both AI and ET phenotypes. The majority of these genes are attributed to the regulation of protein secretion, transport, and localization; however, all enriched genes had ties to fertility and embryonic loss in mammals. These enriched genes included those that have been known to previously affect fertility through the production and heightened sensitivity of essential sex steroids such as growth hormone releasing hormone (*GHRH*) and inhibin beta B (*INHBB*) [39]. While *GHRH* leads to the regulation of growth hormone that is essential for gametogenesis in females, *INHBB* is connected to the number of oocytes that remain in the ovary, as well as connected to the successful conception of pregnancy [39,40]. Genes that are responsible for the protein-coding of these hormones are imperative to study concerning fertility in dairy cattle receiving different reproductive technologies. Many other genes include immune response genes such as the interleukin gene family (*IL1A*, *IL1B*, *IL10*, and *IL18*) and the toll-like receptor genes (*TLR2*, *TLR6*, and *TLR10*); these are important for protection against maternal immune response as well as the increase in uterine natural killer cells that help support embryonic development in early implantation, leading to lower instances of pregnancy loss [41,42,43]. 

## 4. Discussion

The results from the GWAS and the GSEA-SNP provide a foundation for identifying areas of the genome associated with HCR1 for heifers bred by AI or that were ET recipients. The identification of loci associated with HCR1 provides opportunities to predict which cattle are more likely to experience embryonic loss to an AI or ET service, and it also provides insights into potential mechanisms of the loss through the identification of positional candidate genes, enriched gene sets, and leading edge genes. DNA variants that affect these genes may result in the reduced or over-expression of genes that alter embryonic survival. 

In this study, no loci were associated with HCR1 in heifers bred by AI. In comparison, there were three loci associated with HCR1 in heifers that were ET recipients. This lack of association in AI-bred heifers occurred even though there were more AI-bred heifers in this study (*n* = 1575) that conceived to the first service than heifers that served as ET recipients (*n* = 988). Although no loci were shared (because an association was not detected) with HCR1 in AI-bred heifers, there was also a lack of sharing of leading edge genes between AI bred and ET recipients. This suggests that there may be different processes or mechanisms that fail in the establishment of pregnancy in cattle that are ET recipients than in cattle bred by AI. This is supported by previous studies that have reported that the use of ET in cattle can lead to a 10% higher instance of embryonic loss due to elevated body temperature thought to be derived from an elevated immune response from the ET recipient [44]. Other studies did not find a difference between embryonic loss in AI and ET; however, they suggested that in vivo-produced embryos from superovulation are of lower quality and could potentially decrease the successful pregnancies in ET recipients [45]. Overall, many aspects of placentation, embryonic growth, and maternal immune response differ between in vitro-produced and in vivo-derived embryos and their subsequent pregnancy successes, many of which are still under investigation. 

### 4.1. Comparison of Positional Candidate Genes with Other Studies

Loci associated with HCR1 in heifers that were ET recipients were compared to previous studies with diverse fertility phenotypes and cattle breeds (Supplemental Table S1). The locus on BTA6 associated (*p* = 3.8 × 10^−7^) with HCR1 in the recessive model was also associated with the number of times bred (by AI) to maintain a successful pregnancy in an independent Holstein population [29]. This locus contains the protein coding of the actin filament-associated protein (*AFAP1*) gene that has been identified as a predictive biomarker for pre-eclampsia [46,47], as well as being expressed in the uninucleate cells of the bovine placenta at days 17 and 50 of gestation [48]. *AFAP1* has also been previously found to be a leading edge gene for sub-fertile and high-fertile cattle [49].

One positional candidate gene, angiotensin-converting enzyme 2 (*ACE2*), associated with HCR1 in heifers receiving ET on BTAX was previously associated with female fertility. *ACE2* is expressed in ovarian granulosa and theca cells in cattle [50,51], and in the human placenta [52]. The expression of *ACE2* impacts normal pregnancy maintenance by establishing blood pressure patterns during pregnancy that are not detrimental to the dam through angiotensin 1–7 [53,54,55]. Some animal models suggest that *ACE2* deficiency leads to undesirable placenta phenotypes, such as insufficient blood supply, placental lesions, and higher levels of placental inflammation [56,57,58,59]. Studies consisting of *ACE2* knock-out mice concluded that the under-expression of the gene led to lower fetal weight and decreased intrauterine growth mainly due to poor placenta vasculature and abnormal uterine artery remodeling during gestation [60,61]. *ACE2* may also contribute to female fertility during the embryonic establishment of blood supply and development through the first 30 days of gestation in women [62,63]. 

### 4.2. Comparison of Leading Edge Genes with Other Studies

The GSEA-SNP results of the ET recipients had leading edge genes that have a direct connection to fertility and embryonic survivability. One important leading edge gene associated with conception rate to the first service in ET recipients was *GHRH*. The release of GHRH from the hypothalamus leads to the regulation of growth hormone from the pituitary gland, which targets receptors located on the ovary for multiple functions such as steroidogenesis [64]. A study suggested that the presence of growth hormone significantly (*p* < 0.01) increased cleavage rate and blastocyst formation by 12% in bovine oocyte in vitro maturation [60]. 

Another leading edge gene of note is *INHBB*, which codes for the glycoprotein hormone inhibin B that was previously correlated with oocyte reserves and has a negative correlation with circulation follicle-stimulating hormone, as well as a positive correlation with estradiol levels [65]. Lower levels of this hormone are connected to lower pregnancy rates due to poor response to induced ovulation while using assisted reproductive technologies [61,66].

*PKD2* has been identified as a leading edge gene encompassing both artificial insemination (AI) and embryo transfer (ET) services for a higher conception rate to the first service. The observed overlap of *PKD2* across breeding types in dairy cattle further hints at its relevance to fertility beyond varying reproductive technologies. Expression studies reveal high *PKD2* levels during the initial five weeks of embryonic development [67]. Investigations in mice suggest that *PKD2* plays a crucial role in embryonic and placental tissue development, as *PKD2* knockout mice were associated with embryonic lethality, abnormal placental vascularization, issues in smooth muscle organization, and an increased amniotic fluid accumulation [68]

Leading edge genes enriched (NES ≥ 3.0) with HCR1 were compared to genes that were expressed during embryonic development in the placenta of cattle, as reported by Davenport et al. in 2023 [48]. Heifers that received AI services had six leading edge genes (*ATP1A1*, *ATP1B3*, *SCNN1A*, *SLC1A1*, *SLC6A6*, and *SLC8A1*) that were expressed in bovine placental cell clusters at days 17, 24, 30, and 50 of gestation (Table 3). For the ET recipient heifers, there were 26 leading edge genes (*ANXA4*, *APOA1*, *APOA2*, *ARRB1*, *CABP1*, *CASP4*, *CYLD*, *DNM1L*, *F2R*, *FKBP1B*, *GAPVD1*, *HSPA5*, *IFNAR1*, *IL18*, *KDELR1*, *KDELR2*, *OAZ1*, *PDE2A*, *PKIA*, *PRDX1*, *RBP4*, *SNAP25*, *TGFB1*, *TGFB2*, *TLR2*, and *YWHAB*) and a positional candidate gene (*PIR*) for HCR1 that were expressed in bovine placental tissues ([48]; Supplemental Figure S1).

Four HCR1 leading edge genes for heifers receiving ET (*APOA1*, *HSPA5*, *IL18*, and *TLR2*), with the overarching function of protein transport and localization, are associated with recurring embryonic loss in women [69,70,71,72]. The apolipoprotein A-1 (*APOA1*) gene influences the production of estrogen and progesterone, which are crucial for pregnancy [73]. The corpus luteum produces progesterone to maintain pregnancy until this role can be replaced by the production of progesterone by the placenta. The decreasing levels of progesterone allow for levels of estradiol to rise and cyclicity to continue [74]. *APOA1* was expressed in endothelial and mesenchyme cell clusters at various time points in early pregnancy [48]. Expression in these cell types can be connected to the initial start of placentation as the endothelial cells within the uterus go through the mass remodeling and enhanced vascularization needed to supply sufficient blood flow [75]. In cattle, the epithelial cells also have a period of transition where they gain mesenchymal cell characteristics, which is often referred to as epithelial to mesenchymal transition [76,77]. This stage is thought to support a more ideal uterine environment for implantation and placentation, with the cells showing high proliferation and increased immune characteristics to protect the embryo [76]. When the presence of an embryo is not detected, these transitioned mesenchyme cells transition back to epithelial cells, and are commonly associated with the regeneration and proliferation of the uterus [77]. High levels of expressed *APOA1* are associated with implantation failure [67,78,79,80]. High expression levels of *APOA1* serve as a biomarker for poor endometrial environment and as a measure of success in vitro-produced embryos, as 76% of early miscarriage cases had high *APOA1* levels when compared to controls [81]. This downregulation of *APOA1* levels suggests the endometrial preparation of an environment conducive to pregnancy. This conducive environment allows for a lower likelihood of embryonic loss due to implantation failure or inflammation in the uterus, and allowing cattle to have higher chances of conception to the first service [81].

The heat shock protein family member A Hsp70 5 (*HSPA5*) gene (also referred to as *GRP78/BiP*) is a leading edge gene that has been associated with recurrent pregnancy loss in women. The HSPA5 protein resides in the endoplasmic reticulum, where it facilitates cellular protein folding [82]. The *HSPA5* gene is expressed in bovine uninucleate and binucleate cell types during early pregnancy, such as days 17 through 30 of gestation [48]. This expression in both the uninucleate and binucleate cells suggests the importance of the gene in the production, migration, and success of placentation through the increased development of these cell types in early gestation [83,84]. The establishment of the placenta is crucial for cattle to remain pregnant and not suffer from an embryonic loss event. The gene expression of *HSPA5* within the endometrium has been reported as negatively correlated with estrogen levels, and an increase in embryo implantation failure in mice [72,82,85]. The expression of *HSPA5* was detected in the endometrial luminal epithelium cells at day 50 of gestation [48]. This representation of *HSPA5* in the reproductive tissues along with studies suggesting high levels of implantation failure make this gene vital, as high levels of implantation failure are synonymous with high levels of embryonic loss. 

In the embryonic stage of pregnancy, interleukin 18 (*IL18*) is primarily secreted within the endometrium and placenta [86,87]. The secretion of *IL18* within the reproductive tract initiates the expression of tumor necrosis factor alpha and uterine natural killer cells in in vitro fertilization [87,88]. In cattle, the downregulation of *IL18* around day 16 of gestation showed a –2-fold change, suggesting a level of immune system protection in cattle and possibly reducing instances of embryonic loss [41]. This suggested immune response is supported by single-cell data showing expression in macrophage cells in the cattle placenta [48]. That an interleukin would be important in pregnancy establishment and maintenance is supported by studies on interleukin 1 beta (*IL1B*), another associated leading edge gene, and interleukin 15 (*IL15*), for which over-expression leads to miscarriage and implantation failure [89]. Uterine natural killer cells also express another enriched gene toll-like receptor 2 (*TLR2*). This leading edge gene may be involved with embryonic survival through the interaction of uterine natural killer cells at the implantation site and in the placenta [42,43]. *TLR2*, similar to *IL18*, was found to be expressed within the macrophage cells of the placenta [48]. Macrophages and uterine natural killer cells are the most common immune cells present at the placentation site in humans, accounting for up to 90% of all leukocytes [90,91]. While the exact breakdown of the placenta in cattle has not yet been determined, many human studies have focused on the interaction of uterine natural killer cells and macrophages and their functions in successful implantation. The *TLR2* gene is also expressed within the cerebellum during early embryonic development; however, its function within the nervous system is not well understood [92]. 

Leading edge genes enriched for HCR1 in ET heifer recipients and previously associated with fertility in mice and cattle include *CASP4*, *PKD2*, *RBP4*, and *TGFB1*. Caspase 4 (*CASP4*) is expressed in bovine mesenchyme cell clusters at days 17 and 24 of gestation, as well as in placental macrophage cell clusters at days 30 and 50 of gestation [48]. This expression is potentially associated with successful implantation, embryonic development, and maternal immune response [76]. 

The retinol-binding protein 4 (*RBP4*) gene is expressed in the mesenchymal cell cluster of the developing bovine placenta across gestation [48]. Uninucleate cells make up 80% of the trophoblast in the chorionic villi, with the other 20% being binucleate cells [93]. Binucleate cells are more well-known, as they fuse to the uterine epithelium during implantation, transport molecules from the fetus to the mother, as well as aiding in steroidogenesis [94,95,96]. The regeneration and presence of binucleate cells are essential to the maintenance of pregnancy in cattle and other ruminants as they establish the fetal–maternal complex [96]. *RBP4* is associated with conceptus elongation, HCR1, cow conception, and daughter pregnancy rate in Holstein heifers [97,98]. The onset of conceptus elongation resulted in a 94% increase in *RBP4* expression [99]. It is well known that implantation and conceptus elongation are processes prone to failure within dairy cattle pregnancy establishment [100], making this gene a good candidate for further research regarding its role in embryonic loss. 

Transforming growth factor beta 1 (*TGFB1*) has an array of functions, especially within the reproductive tract [101]. *TGFB1* is differentially upregulated by a 6.7 log2-fold change between day 17 and day 24 of gestation in uninucleate cells [48]. The regeneration of binucleate cells by uninucleate cells is essential to the establishment and maintenance of pregnancy, and therefore implies the necessity of these cell types for embryonic survivability [95,96]. The *TGFB* family of genes is essential to immunoregulation during pregnancy, embryo implantation, and placental development [101,102]. In particular, *TGFB1* is connected to embryonic loss, with one study reporting up to 100% embryonic loss at the pre-implantation stage (day 2–3 of gestation) of pregnancy when the gene was knocked out in mice [103]. In addition, 80% of *TGFB1* null mice displayed embryonic loss due to implantation failure [104]. While the actual role of *TGFB1* in relation to pregnancy loss in cattle is not fully appreciated, more investigation is warranted to identify the mechanisms associated with *TGFB1* in embryonic loss. 

## 5. Conclusions

The GWAS identified no loci associated with HCR1 in AI-bred heifers, and three loci and four positional candidate genes associated with HCR1 in heifers that were ET recipients. The GSEA-SNP identified one gene set and 15 leading edge genes enriched for HCR1 in AI-bred heifers, and four gene sets and 142 leading edge genes enriched for HCR1 in Holstein heifers that were ET recipients. As with the results from the GSEA-SNP, there are clearly differences between genomic regions associated or enriched with HCR1 in heifers bred by AI and those that were ET recipients. The genomic results are consistent with reports of the physiological differences that cause embryonic loss in AI and ET pregnancies. Overall, one locus on BTA6 associated with HCR1 in ET recipients was validated in previous cattle studies to be associated with the number of times inseminated to successful pregnancy in Holsteins [29]. The 26 leading edge genes associated with HCR1 ET recipients that were supported by single-cell data also validate these genes’ presence during the embryonic stage of gestation in cattle [48]. Additional support for the genomic findings is provided by the fact that many of the positional candidate and leading edge genes have been associated with embryonic loss in mice and humans.

Many of the discovered genes associated with embryonic loss events of heifers receiving AI or ET encompassed functions of hormone production, hormone regulation, and immune response. Normal hormone secretion and function is essential for the maintenance of pregnancy [39,40,94]. Genes associated with immune response are also essential to embryonic survivability, as many allow for protection from the maternal aspect that can lead to embryonic death [41,42,43]. It is also imperative that gene expression not only protect the developing embryo, but also allow for the development of the innate immune response [105]. 

In Holstein dairy cattle, embryonic loss is a common reason for culling causing producers’ substantial financial loss. Identifying regions of the genome that are associated with embryonic loss in heifers bred by AI or for heifers that are ET recipients allows for the potential use of genomic selection in making selection and breeding decisions. This study suggests that one locus and 44 genes are important in embryonic loss and could be used for the selection for HCR1.

## Figures and Tables

**Table 1:** Summary of conception rate to the first service for Holstein heifers bred by artificial insemination and heifers that were embryo transfer recipients and remained pregnant.

Breeding Type	No. Pregnant to the First Service	No. Not Pregnant to the First Service	Total
Artificial insemination	1575	1254	2829
Embryo Transfer Recipients	988	1098	2086

**Table 2:** Single-nucleotide polymorphisms associated with HCR1 for ET recipients.

BTA ^1^	Position (bp) ^2^	*p*-Value ^3^	SNP ^4^	Minor Allele	Freq. Cases ^5^	Freq. Controls
6	114,186,062	3.81 × 10^−7^	rs136769131	A	0.379	0.433
13	83,298,964	6.63 × 10^−6^	rs43097654	T	0.050	0.027
X	127,944,565	2.47 × 10^−6^	rs133237751	T	0.392	0.373
X	127,962,670	1.78 × 10^−6^	rs110097934	A	0.391	0.373
X	127,976,137	1.78 × 10^−6^	rs110705866	T	0.475	0.409

^1^ *Bos taurus* chromosome location of the locus. ^2^ Location in chromosome base pairs of the significant SNP. ^3^ Significance (*p*-value) of the most significant SNP associated with HCR1 and ET. ^4^ The significant SNP associated with HCR1 to ET recipients listed by the rs number assigned by the National Center for Biotechnology Information SNP database “https://www.ncbi.nlm.nih.gov/snp/ (accessed on 30 April 2024)”. ^5^ When case frequency > control frequency it is the unfavorable allele.

**Table 3:** Enriched gene sets and leading edge genes with conception rate to the first service in Holstein heifers.

Gene Set (Database ID) ^1^	# Genes ^2^ (# LEG) ^3^	NES ^4^	Leading Edge Genes ^5^
Heifer Conception Rate to the First AI Service			
Sodium Ion Transmembrane Transporter Activity (GO:0015081)	25 (13)	3.0	*SCN4B*, *ATP1B3*, *SLC1A1*, *SCNN1A*, *SLC6A3*, ***PKD2***, *ATP1A1*, *SLC24A1*, *SLC6A17*, *SLC6A15*, *SLC8A1*, *SLC6A13*, *SLC6A6*
Heifer Conception Rate to the First ET Service			
Regulation of Protein Transport (GO:0051223)	121 (49)	3.4	***F2RL1***, ***F2R***, ***DRD2***, ***KDELR1***, ***KDELR2***, ***IL5***, ***HSPA5***, ***HMGN3***, ***PDE2A***, ***PIK3R1***, ***GCG***, ***TGFB2***, ***TRIP6***, ***FIS1***, ***GAPVD1***, ***ZPR1***, ***TLR2***, ***OAZ1***, ***DNM1L***, ***YWHAB***, ***CASP4***, ***TLR10***, ***TLR6***, ***CDK5***, ***CABP1***, ***PKD2***, ***CYLD***, ***CLEC6A***, ***SLC51B***, ***PBLD***, ***TGFBR1***, ***ASPH***, ***ARRB1***, ***IL18***, ***NOD2***, ***GPLD1***, ***SIRT4***, ***ANG***, ***PKIA***, ***ANKRD1***, ***PRDX1***, ***IL1B***, ***DMTN***, ***SERGEF***, ***APOA1***, ***IFNG***, ***TGFB1***, ***RBM22***, ***APOA2***
Regulation of Establishment of Protein Localization (GO:0070201)	125 (53)	3.23	***F2RL1***, ***F2R***, ***DRD2***, ***KDELR1***, ***KDELR2***, ***IL5***, ***HSPA5***, ***HMGN3***, ***PDE2A***, ***PIK3R1***, ***GCG***, ***TGFB2***, ***TRIP6***, ***FIS1***, ***GAPVD1***, ***ZPR1***, ***TLR2***, ***OAZ1***, ***DNM1L***, ***YWHAB***, ***CASP4***, ***TLR10***, ***TLR6***, ***CDK5***, ***CABP1***, ***PKD2***, ***CYLD***, ***CLEC6A***, ***SLC51B***, ***PBLD***, ***TGFBR1***, ***ASPH***, ***ARRB1***, ***IL18***, ***NOD2***, ***GPLD1***, ***SIRT4***, ***ANG***, ***PKIA***, ***ANKRD1***, ***PRDX1***, ***IL1B***, ***DMTN***, ***SERGEF***, ***APOA1***, ***IFNG***, ***TGFB1***, ***RBM22***, ***APOA2***, ***ANXA4***, *APOD*, *SNAP25*, *RBP4*
Liposaccharide Metabolic Process and Glycolipid Metabolic Process (GO:1903509)	18 (5)	3.05	*DPM3*, *ST6GALNAC6*, *BAX*, *PIGY*, *GPLD1*
Regulation of Protein Secretion (GO:0050708)	69 (35)	3.03	***F2RL1***, ***F2R***, ***DRD2***, ***IL5***, ***HMGN3***, ***GCG***, ***TGFB2***, ***TLR2***, ***DNM1L***, ***CASP4***, ***TLR10***, ***TLR6***, ***CLEC6A***, ***ARRB1***, ***NOD2***, ***GPLD1***, ***SIRT4***, ***ANG***, ***ANKRD1***, ***IL1B***, ***SERGEF***, ***APOA1***, ***IFNG***, ***TGFB1***, ***APOA2***, ***ANXA4***, *RBP4*, *FKBP1B*, *IL10*, *IL1A*, *NR1H3*, *IFNAR1*, *GHRH*, *GHRL*, *INHBB*, *CHGA*

^1^ Database sources. GO = Gene Ontology. ^2^ Total number of genes in the gene set. ^3^ Number of leading edge genes (LEGs) associated with HCR1 in the gene set. ^4^ NES is the normalized enrichment score. ^5^ Gene names of leading edge genes. Leading edge genes in bold are shared in more than one gene set.

## Data Availability

The original contributions presented in the study are included in the article/Supplementary Materials, further inquiries can be directed to the corresponding author.

## Supplementary Materials

The following supporting information can be downloaded at: https://www.mdpi.com/article/10.3390/genes15060765/s1, Table S1: Cattle fertility studies that were compared with the current study to identify shared loci, positional candidate genes or leading edge genes [106,107,108,109,110,111,112,113,114,115,116,117,118,119,120,121,122,123,124,125,126,127]; Figure S1: Bovine placenta single-cell gene expression plots [48] of leading edge genes associated with HCR1 in ET-recipient heifers.
