# Supplementary material for: Identifying Regions of the Genome Associated with Conception Rate to the First Service in Holstein Heifers Bred by Artificial Insemination and as Embryo Transfer Recipients
Source: Genes (Basel). 2024 Jun 11;15(6):765. doi: 10.3390/genes15060765 (PMC11202900; doi:10.3390/genes15060765)
Supplement: Supplementary file 1 [file genes-15-00765-s001.zip › genes-3034965-supplementary.pdf]

Table S1. Cattle fertility studies that were compared with the current study to identify shared loci, positional candidate genes or leading-edge genes.

Previous Study	External Study Phenotype	External Study Breed
Akanno et al., 2015 [106]	Pregnancy Rate	Canadian crossbred beef heifers
Blaschek et al., 2011 [107]	Conception Rate in Bulls	Holsteins
Cochran et al., 2013 [108]	Heifer Conception Rate<br>Cow Conception Rate	Canadian Holsteins
Cole et al 2011 [109]	Daughter Pregnancy Rate	Holsteins
Cole et al., 2020 [110]	Haplotype tests for economically important traits	Holsteins
Davenport et al., 2023 [47]	Single Cell of the Bovine Placenta	Holsteins
Galliou et al., 2020 [29]	Conception Rate to the First Service in Heifers<br>Times Bred to Successful Pregnancy in Heifers	Holsteins
Guillaume et al., 2007 [111]	Non-return Rate at days 28, 56, 90 and 282	French Holsteins
Häfliger et al., 2022 [112]	Haplotypes for Deficient Homozygosity	Switzerland Holsteins
Höglund et al, 2009 [113]	Interval From First to Last Insemination<br>Non-return Rate at 56 days	Denmark Holsteins<br>Swedish Holsteins

	Number of inseminations per conception based on Sire	
Höglund et al., 2014<br>[114]	Number of Inseminations per Conception Heifers<br>Number of Inseminations per Conception Cows	Nordic Holsteins
Höglund et al., 2015<br>[115]	Number of inseminations per conception	Nordic Red Holsteins
Huang et al., 2010<br>[116]	Fertilization Rate<br>Blastocyst Rate<br>IVF Success	Holsteins
Iso-Touru et al. 2016<br>[117]	Milk Production and Fertility	Nordic Red Holsteins
Liu et al., 2017<br>[118]	Conception Rate at First Insemination Heifers<br>Conception Rate at First Insemination Cows	Nordic Holstein<br>Chinese Holstein
Minnozi et al., 2013<br>[119]	Days to First Service<br>Aggregate Fertility Index<br>Calving Interval<br>Non-return Rate	Italian Holsteins
Minten et al., 2013<br>[120]	High fertile Vs Low Fertile Cattle based on Embryo Transfer	Crossbred beef heifers
Moraes et al., 2018<br>[121]	Differentially Expressed Genes in High and Low Fertile Heifers<br>Differentially Expressed Genes in Open and Pregnant Heifers	Angus Heifers

Müller et al., 2017<br><br>[122]	FSTC in Cows and Heifers	Holsteins
Nayeri et al., 2016<br><br>[123]	Calving to First Service<br><br>Days Open	Canadian Holsteins
Neupane et al., 2017<br><br>[36]	High fertile Vs Sub-fertile Cattle based on Embryo Transfer	Crossbred beef heifers
Ortega et al., 2016<br><br>[124]	Heifer Conception Rate<br><br>Cow Conception Rate<br><br>Daughter Pregnancy Rate	Holsteins
Ortega et al., 2017<br><br>[125]	Days Open<br><br>Pregnancy Rate at the First Service<br><br>Services per Conception	Holsteins
Sahana et al., 2010<br><br>[126]	Interval from calving to first insemination<br><br>Number of inseminations per conception<br><br>Days from last insemination	Danish Holstein<br><br>Swedish Holsteins
Wu et al., 2019<br><br>[127]	Haplotypes of Early Embryonic Lethality	Nordic Holsteins

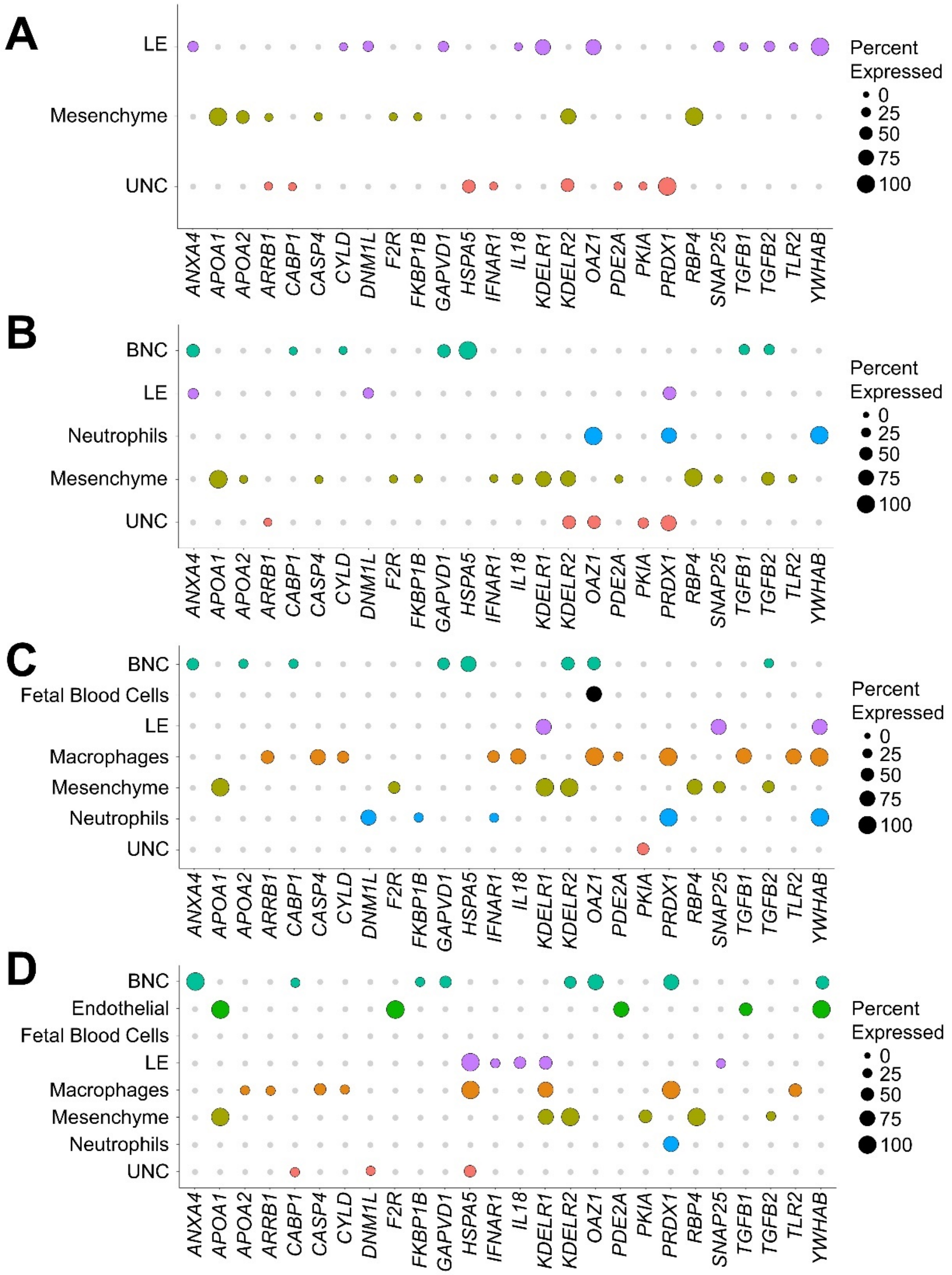

Figure S1. Bovine placenta single cell gene expression plots [47] of leading edge genes enriched with HCR1 in ET recipient heifers. Expression of genes in different placental cell types at days 17 (panel A), 24 (panel B), 30 (panel C) and 50 (panel D) days of gestation. Placental cell types consisted of binucleate cells (BNC), endothelial cells, fetal blood cells, endometrial luminal epithelial cells (LE), macrophages, mesenchyme neutrophils and uninucleate cells (UNC).
